# Multigene prognostic tests in breast cancer: past, present, future

**DOI:** 10.1186/s13058-015-0514-2

**Published:** 2015-01-27

**Authors:** Balázs Győrffy, Christos Hatzis, Tara Sanft, Erin Hofstatter, Bilge Aktas, Lajos Pusztai

**Affiliations:** MTA TTK Lendület Cancer Biomarker Research Group, Magyar tudósok körútja 2, Budapest, H-1117 Hungary; MTA-SE Pediatrics and Nephrology Research Group, Bókay u. 53, Budapest, H-1083 Hungary; Second Department of Pediatrics, Semmelweis University, Tűzoltó u. 7-9, Budapest, H-1094 Hungary; Yale Cancer Center, Yale School of Medicine, 333 Cedar Street, PO Box 208032, New Haven, CT 05620 USA

## Abstract

There is growing consensus that multigene prognostic tests provide useful complementary information to tumor size and grade in estrogen receptor (ER)-positive breast cancers. The tests primarily rely on quantification of ER and proliferation-related genes and combine these into multivariate prediction models. Since ER-negative cancers tend to have higher proliferation rates, the prognostic value of current multigene tests in these cancers is limited. First-generation prognostic signatures (Oncotype DX, MammaPrint, Genomic Grade Index) are substantially more accurate to predict recurrence within the first 5 years than in later years. This has become a limitation with the availability of effective extended adjuvant endocrine therapies. Newer tests (Prosigna, EndoPredict, Breast Cancer Index) appear to possess better prognostic value for late recurrences while also remaining predictive of early relapse. Some clinical prediction problems are more difficult to solve than others: there are no clinically useful prognostic signatures for ER-negative cancers, and drug-specific treatment response predictors also remain elusive. Emerging areas of research involve the development of immune gene signatures that carry modest but significant prognostic value independent of proliferation and ER status and represent candidate predictive markers for immune-targeted therapies. Overall metrics of tumor heterogeneity and genome integrity (for example, homologue recombination deficiency score) are emerging as potential new predictive markers for platinum agents. The recent expansion of high-throughput technology platforms including low-cost sequencing of circulating and tumor-derived DNA and RNA and rapid reliable quantification of microRNA offers new opportunities to build extended prediction models across multiplatform data.

## Introduction

**Figure 1:**
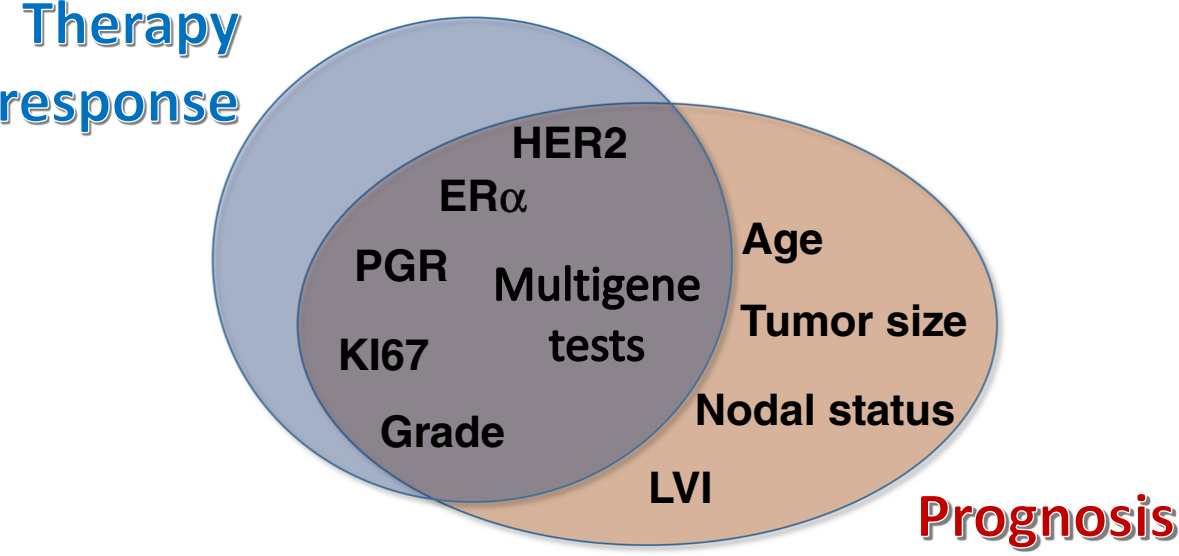
**Prognostic and predictive relationship between multigene signatures and prognostic and predictive features in breast cancer.** ER, estrogen receptor; HER2, human epidermal growth factor receptor 2; LVI, lymphovascular invasion; PR, progesterone receptor.

Oncologists involved in the clinical management of breast cancer have to consider several different clinical and molecular characteristics of the tumor, in addition to patient preferences and comorbidities, when formulating therapeutic recommendations for early stage, potentially curable cancers. Some clinical–pathologic characteristics including tumor size, nodal status, and lymphovascular invasion are risk factors associated with prognosis (that is, the probability of disease-free survival with surgery alone in the absence of any systemic adjuvant therapy), while others such as histologic grade, estrogen receptor (ER), progesterone receptor (PR), and human epidermal growth factor receptor 2 (HER2) status, and proliferation rate are associated with both prognosis and sensitivity to treatment modalities (Figure 1) [1]. Low histologic grade, ER-positive status, and HER2-negative status are each independently associated with better prognosis in patients treated with surgery alone (or with surgery and adjuvant endocrine therapy if ER-positive) and also predict lesser sensitivity to chemotherapy. On the contrary, high histologic grade and high proliferation rate are associated with worse prognosis but at the same time also predict for higher chemotherapy sensitivity, which is apparent from the higher rates of pathologic complete response to neoadjuvant chemotherapy and higher relative benefit from adjuvant chemotherapy among cancers with these molecular features.

There are a number of subtleties to predictive and prognostic markers that clinicians and investigators need to be aware of. The prognostic or predictive strength of different features is variable. For example, nodal status is a more powerful prognostic predictor than HER2 status [2]. The same marker can have different prognostic or predictive values in different molecular subtypes of breast cancer. For example, high proliferation rate measured by Ki67 expression and high grade have greater prognostic value and are more predictive of chemotherapy response in ER-positive cancers than in triple-negative breast cancers (TNBCs) (ER-, PR- and HER2-negative). All currently known prognostic and predictive features are only partially independent from one another. For example, ER-negative cancers tend to have high grade and high proliferation rate. Larger cancers are more likely to be node-positive. Finally, the different markers represent different types of distributions and are quantified with variable accuracy. Age, tumor size and Ki67 expression represent continuous variables that approximate a normal distribution. The ER and PR expression by immunohistochemistry or by mRNA levels are continuous variables with a bimodal distribution. Histologic grade and nodal status are ordinal variables, whereas HER2 gene amplification results are used as a binary variable. The quantification of ER, PR and HER are increasingly standardized and between-laboratory reproducibility has improved substantially over the past few years. However, histologic grade and Ki67 immunohistochemistry has only modest between-laboratory concordance. Tumor size measurements in many pathology laboratories tend to cluster around whole numbers, suggesting rounding to the nearest number.

When multiple, partially independent factors measured with variable accuracy on different scales are associated with an outcome, the most accurate predictions can only be achieved by multivariate prediction models. This justifies the efforts to build multivariate prognostic models such as AdjuvantOnline (Adjuvant! Inc., San Antonio, TX, USA) and multigene predictors. Empirically developed multigene prognostic predictors have the theoretical advantages of optimal use of information from continuous variables, proper weighting of each variable, and robustness through redundancy by capturing similar information from multiple genes (that is, ER activity or proliferation are assessed through a cluster of genes rather than ER or Ki67 alone) and the potential to identify and incorporate new molecular variables into the model. However, they also have limitations; most importantly, some powerful anatomical–pathologic prognostic risk factors such as tumor size and nodal status have no consistent molecular imprint and therefore these variables are not captured by empirically developed gene signatures. Also, some of the theoretical advantages have not fully materialized in practice. No robust, new, prognostic genes have been identified that are unrelated to proliferation or ER signaling, and when novel genes are included in multigene tests their contribution to the outcome prediction is modest. To what extent quantification of ER-related or proliferation-related metagenes provides robustness or increased accuracy over reliably quantifying ER and Ki67 also remains uncertain. Despite these limitations, there is growing consensus that multigene prognostic gene signatures provide standardized, complimentary information to routine pathological variables including tumor size, nodal status and histologic grade. Multigene prognostic assays are now endorsed by the American Society of Clinical Oncology, St. Gallen and National Comprehensive Cancer Network guidelines as information that could assist therapeutic decision-making in ER-positive cancers. The following sections are brief reviews of clinically available assays.

## IHC4 assay

The IHC4 assay is based on a multivariate model that uses semiquantitative information from immunohistochemical assessment of ER, PR, HER2 and Ki67. The assay is performed on formalin-fixed, paraffin-embedded (FFPE) tumor biopsy specimens and a risk score for recurrence is calculated by an algorithm [3].

While the IHC4 test is elegant in its simplicity and has similar performance characteristics to the more complex and expensive commercial tests, it uses information from ER, PR and Ki67 differently to how these markers are currently interpreted in routine practice. Physicians tend to use these markers as binary categories (that is, ER-positive vs. ER-negative, Ki67 low vs. Ki67 high, and so forth) and create distinct groups (with four binary markers, 16 different marker groups are possible); in contrast, IHC4 uses a mathematical formula that weighs the semiquantitative expression values and combines these into a single risk score. The mathematical equation behind the IHC4 score is public; however, in the absence of standardized quantification of each of the four variables that would match the IHC assay sensitivity and the dynamic ranges used in the seminal paper, applying the formula to local pathology results could be highly misleading.

## MammaPrint score

MammaPrint (Agilent, Amsterdam, the Netherlands) is a microarray-based prognostic score performed by a central laboratory that was approved by the US Food and Drug Administration and by regulators in the European Union as an adjunct prognostic assay for breast cancer patients younger than 61 years of age with stage I/II, lymph node-negative or one to three lymph node-positive disease [4].

MammaPrint measures the mRNA expression of 70 genes and stratifies patients into low-risk or high-risk prognostic groups [5]. The prognostic risk discrimination is good among ER-positive cancers but almost all ER-negative cancers are classified as high risk, which limits the score’s clinical value in this disease subset. Retrospective analysis of a large multicenter patient cohort (*n* = 541) suggested that only the high-risk ER-positive patients benefited from adjuvant chemotherapy [6,7]. The original assay required fresh-frozen material, which limited its clinical uptake, particularly in the United States. More recent versions of the test can use FFPE tissues [8].

A large prospective trial (MINDACT) has tested the clinical utility of MammaPrint and accrued 6,600 patients between 2006 and 2011; results from this trial are expected in 2015. This study has also created a unique tissue and gene expression data resource that will allow the development and testing of the next generation of prognostic gene signatures.

## PAM50/risk of recurrence/Prosigna kit

In September 2013 the US Food and Drug Administration approved a second prognostic signature (Prosigna; Nanostring Technologies, Seattle, WA, USA) that also received clearance in the European Union. Prosigna is a diagnostic kit that uses Nanostring technology to quantify mRNA expression of 50 genes used in the PAM50 molecular classification algorithm and a series of housekeeping genes (that are used for normalization) as well as positive and negative controls in FFPE tissues to compute a risk of recurrence score. The risk of recurrence score reflects but does not explicitly report the intrinsic breast cancer subtype of the case [9,10].

The assay is approved to estimate distant recurrence-free survival for stage I/II (including one to three positive nodes), ER-positive breast cancer in postmenopausal women treated with adjuvant endocrine therapy. An appealing aspect of this test is that it can be performed by local pathology laboratories although it requires an expensive piece of equipment, the Nanostring nCounter Dx Analysis System (Nanostring Technologies).

## Oncotype DX assay

Currently, Oncotype DX (Genomic Health, Redwood City, CA, USA) is the most widely used prognostic assay for ER-positive cancers in the United States. This assay utilizes expression measurements from 16 cancer-related genes and five housekeeping genes to compute a recurrence score from 0 to 100, which can be categorized into low-risk (score <18), intermediate-risk (score 18 to 30) or high-risk (score ≥31) groups [11]. Measurement is performed on FFPE specimens in a central laboratory.

Several studies on archived tissues from randomized adjuvant chemotherapy trials demonstrated benefit from adjuvant chemotherapy primarily in patients with high recurrence score. A larger, prospective, randomized clinical trial (TAILORx) has tested the utility of adjuvant chemotherapy in node-negative woman with intermediate risk for recurrence by Oncotype DX (that is, recurrence score 11 to 25); accrual has been completed and the first results are expected in 2017.

Recently, a multivariate model has been developed that takes information from the Nottingham grade, ER and PR immunohistochemistry results, HER2 status, tumor size and Ki67 expression to predict the recurrence score category [12]. The equation has been tested on large datasets, and through the combination of these routinely available variables all non-intermediate recurrence-score risk categories could be predicted with high accuracy (96 to 100%). This recurrence score category predictor is available online [13].

## Genomic grade index

The Genomic Grade Index (MapQuant Dx, Ipsogen, France) is a microarray-based assay that measures the expression of 97 genes to assign a molecular grade. The assay was developed by comparing gene expression profiles of grade I and grade III tumors [14]. A smaller six-gene version has also been developed that uses RT-PCR technology and can be readily applied to FFPE samples [15]. The test can reclassify histologically intermediate-grade ER-positive cancers into high or low molecular grade with significantly different prognosis.

## Breast cancer index

The Breast Cancer Index (BioTheranostics, San Diego, CA, USA) is an RT-PCR-based assay that can be applied to FFPE tissues and is performed by a central laboratory to predict the risk of distant recurrence in ER-positive, lymph node-negative breast cancers. This assay includes two independent biomarkers, the HOXB13:IL17BR ratio and a five-gene molecular grade index that primarily consists of proliferation-related genes [16,17]. The two signatures together comprise the Breast Cancer Index score.

A strength of the Breast Cancer Index is that it can be used to predict risk of both early (within 5 years) and late (>5 years) distant recurrences and also reports the likelihood of benefit from extended endocrine therapy. The Breast Cancer Index outperformed the IHC4 or Oncotype DX assays in predicting late recurrence when all three tests were applied to the same cases with long-term follow-up [18]. The Breast Cancer Index identifies a substantial minority of patients (35 to 45%) who are at high risk for late recurrences, and these patients may be considered optimal candidates for extended adjuvant therapy.

## EndoPredict assay

The EndoPredict test (Sividon Diagnostics GmbH, Koln, Germany), also an RT-PCR-based assay, measures the expression of eight cancer genes and three housekeeping control genes to stratify patients with ER-positive cancer into a low or a high risk of recurrence if treated with adjuvant endocrine therapy alone [19]. The assay is marketed in Europe as a diagnostic kit that can be performed by local laboratories. In addition, the EndoPredict score has also been combined with nodal status and tumor size to compute a comprehensive risk score termed EPclin. The performance of EPclin was validated in two randomized phase III trials [20]. EndoPredict also identifies ER-positive patients who are at risk for late recurrence [21].

## Concordance of risk assignment by different tests

The advent of multiple different tests available in the clinic for the same purpose inevitably raises the question of concordance in risk assignment when more than one of the tests are applied to the same specimen. Comparative studies indicate that discordant risk prediction frequently occurs when different prognostic assays are applied to the same case. When six genomic signatures, including PAM50/risk of recurrence, MammaPrint, and Oncotype DX, were tested on the same patient cohort, each test had significant prognostic value but individual risk assignments were often discordant [22].

In another study, when molecular classification with PAM50 was compared with Oncotype DX risk categories among the luminal A cancers by PAM50, 70% were low risk and the remainder were intermediate risk by Oncotype DX. Among luminal B cancers, 33% were high risk and 48% were intermediate risk by Oncotype DX. Ninety percent of high-risk cases by Oncotype DX were classified as luminal B and 83% of low-risk cases were luminal A. Importantly, one-half of intermediate recurrence score cancers were recategorized as low-risk luminal A cancers by PAM50 [23].

Another analysis of six different prognostic signatures applied to the same cases also showed only moderate agreement between prediction results in pairwise comparisons; Cohen’s kappa ranged from 0.236 to 0.701 [24]. Similarly high discordance rates for Oncotype DX and MammaPrint risk categories were reported; more than 30% of MammaPrint high-risk cases were reclassified as low risk by Oncotype DX [24].

## Prediction of chemotherapy benefit

It is possible to predict general chemotherapy sensitivity by capturing proliferation-related markers, particularly among ER-positive cancers. The higher chemotherapy sensitivity is reflected by higher pathologic complete response rates to a broad range of preoperative regimens and also by the greater benefit from adjuvant chemotherapy in general. Biomarker subset analysis of two separate studies (NSABP B-20 and SWOG 8814) has shown that adjuvant chemotherapy improved disease-free survival only in ER-positive cancers with high recurrence score, while benefit was not seen, or was minimal, in low-risk and intermediate-risk patients, respectively [14,25].

However, the quest for drug-specific or treatment regimen-specific predictive markers remains elusive. The most plausible explanation for the limited predictive values of proposed molecular predictors for individual drugs is the multifactorial nature of chemotherapy sensitivity. Empirical development of predictive models assumes there are shared molecular features that are common to all, or most, sensitive or resistant cases. If such informative features exist, they can easily be identified through statistical methods and combined into a multivariate model to predict response in future cases. However, it appears that there are very few molecular features which are associated with individual drug response and are shared across many patients [26]. Data from gene expression profiling as well as from next-generation sequencing indicate that each cancer contains a variable number and unique assortment of genomic abnormalities. This suggests that each cancer may be sensitive or resistant to a particular treatment in its own unique way. In other words, a particular biological pathway that contributes to drug sensitivity can be affected through many different mechanisms, including mutations, amplifications, deletions, epigenetic regulation, microRNA, and so forth, at many different levels along the pathway. The increasing recognition of within-tumor cellular and genomic heterogeneity further increases complexity.

A corollary of tumor heterogeneity is that some genome-wide metric of tumor heterogeneity or genomic disturbance may function as biomarkers. Indeed, one of the promising, novel, drug class-specific predictors includes quantifying the effects of homologous DNA recombination deficiency on the genome, regardless of its molecular etiology, by simultaneously assessing whole genome loss of heterozygosity, telomeric allelic imbalance and large-scale state transitions. These three metrics can be combined into a single homologous recombination deficiency score (HRD assay; Myriad Genomic, Salt Lake City, UT, USA). A high homologous recombination deficiency score indicates defective homologous recombination repair that renders cells vulnerable to accumulation of DNA damage after exposure to DNA crosslinking drugs, particularly platinum agents. Several retrospective correlative studies showed a good correlation between the homologous recombination deficiency score and the response to platinum therapy in breast cancer and ovarian cancer [27,28]. The assay is performed on DNA extracted from FFPE tissue sections and uses single nucleotide polymorphism arrays and high-throughput sequencing. Prospective trials to assess the clinical value of the assay for patient selection to platinum therapy are underway.

## Conclusions

Multigene signatures introduced an important concept into prognostic marker research: the need for multivariate prediction models. This represents a conceptual advance over arbitrary groupings based on combinations of single prognostic variables such as clinical tumor staging using the tumor, node, metastasis classification. A second important conceptual contribution was shifting the emphasis from pure, primary prognostic prediction (that is, who has excellent prognosis with surgery alone) to secondary risk stratification, recognizing that essentially all ER-positive breast cancer cases are offered adjuvant endocrine therapy and therefore the relevant clinical question is who remains at high risk for recurrence despite endocrine therapy and is therefore a potential candidate for adjuvant chemotherapy.

The first generation of multigene prognostic predictors was developed empirically by comparing gene expression data from patients who did or did not experience recurrence. These attempts identified ER-related genes and proliferation markers as the two most powerful molecular processes associated with outcome. Since ER has a very broad transcriptional footprint and cell proliferation requires the coordinated expression of hundreds of genes, a very large number of nominally different but equally good prognostic models can be built from the same dataset. This redundancy in the prognostic variable space for ER-positive cancers explains the existence of many different prognostic gene signatures in the commercial space and in academic research laboratories. Since each model uses different gene sets and was optimized in distinct training sets, when multiple models are applied to the same independent validation set they all identify low-risk and high-risk cohorts but also tend to show substantial discordance (20 to 30%) in risk assignment at the individual case level. Somewhat surprisingly, no molecular markers emerged that are associated with tumor size and nodal status, which are the two most important anatomical prognostic variables. This also implies that prognostic information embedded in size and nodal status is not captured in empirically developed prognostic gene signatures. For this reason, multivariate clinical prognostic models such as AdjuvantOnline and molecular models tend to result in highly discordant risk assignment at the individual case level.

**Figure 2:**
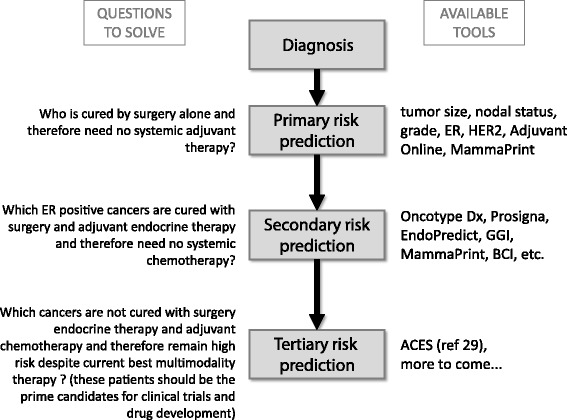
**Conceptual framework for risk stratification and currently available prognostic and predictive tools.** Aces, Adjuvant chemotherapy and endocrine therapy sensitivity signature [29]; BCI, Breast Cancer Index; ER, estrogen receptor; GGI, Genomic Grade Index; HER2, human epidermal growth factor receptor 2.

From these basic performance characteristics emerges the current clinical utility of genomic prognostic assays. All major practice guidelines endorse molecular assays to aid prognostic risk prediction in ER-positive, T1–T2 breast cancers with zero to three positive nodes. While the molecular assays retain their prognostic discriminating value regardless of anatomical risk factors, the final risk of recurrence is determined by both molecular and anatomical features because tumor size and nodal status represent independent prognostic variables [29]. The inverse relationship between proliferation and prognosis and chemotherapy sensitivity in ER-positive cancers conveniently allows the identification of patients who are higher risk for recurrence and at the same time have higher sensitivity to chemotherapy. The flip side of this association is that patients with molecularly low-risk cancers with anatomically high-risk features (for example, multiple positive lymph nodes) may not derive significant benefit from adjuvant chemotherapy – this concept is currently being tested in a prospective randomized clinical trial (SWOG 1007). Genomic prognostic tests are also increasingly used as patient selection tools for clinical trials and to define clinically relevant patient populations for drug development (Figure 2).

Important new areas of research have also emerged. One area where the performance of existing tests could be improved is more accurate prediction of late recurrences in ER-positive cancer. The clinical importance of this is underscored by the recognition that around 50% of all recurrences in ER-positive cancers develop after 5 years and the rate of late recurrences can be reduced by extended adjuvant endocrine therapy. A series of new prognostic tests are emerging (Breast Cancer Index, EndoPredict, Prosigna) that address this diagnostic niche. Another prognostic challenge is to identify patients among the currently high-risk ER-positive cancer cases (high recurrence score or luminal B molecular class) that are no longer at high risk after receiving adjuvant chemotherapy [30]. This represents the next logical extension of residual risk prediction. The clinical importance of a postchemotherapy and postendocrine therapy, tertiary risk predictor is that it could define the ER-positive patient population to whom new drug development should be targeted.

One of the most puzzling observations in breast cancer biomarker research has been the difficulty to identify clinically useful molecular prognostic markers for TNBC. While it is possible to define better and worse outcome groups among these cancers by capturing information about immune cell and inflammatory infiltration, the practical value of these observations is limited because even “good-risk” patients have close to 20% risk of distant recurrence in the absence of systemic adjuvant therapy [31]. Considering the clinical context in TNBC, which involves choosing between observation versus adjuvant chemotherapy, and the lack of data to support that adjuvant chemotherapy could not improve the outcome of the “good-risk” patients further, a 20% risk of recurrence is too high for most patients and physicians. One might propose that searching for pure primary prognostic markers in TNBC is likely to be futile. On the contrary, developing residual risk predictors that identify TNBC with low risk of recurrence after completing adjuvant chemotherapy could be helpful [32]. However, this information can be readily obtained by administering the intended adjuvant chemotherapy preoperatively. Pathologic complete response in this disease subset identifies patients who have excellent long-term survival with a given chemotherapy [33]. The most pressing clinical need for TNBC might be to develop more effective new drugs.

Finally, the advent of several new high-throughput technology platforms including RNA sequencing, microRNA profiling and analysis of free circulating DNA allows substantial broadening of the variable space for multivariate model development. Research groups are exploring these new opportunities to build extended prediction models across multiplatform data.

## Abbreviations

ER: Estrogen receptor
FFPE: Formalin-fixed, paraffin-embedded
HER2: Human epidermal growth factor receptor 2
PR: Progesterone receptor
TNBC: Triple-negative breast cancer
